# Genomic Polymorphism of the Pandemic A (H1N1) Influenza Viruses Correlates with Viral Replication, Virulence, and Pathogenicity *In Vitro* and *In Vivo*

**DOI:** 10.1371/journal.pone.0020698

**Published:** 2011-06-15

**Authors:** Lili Xu, Linlin Bao, Jianfang Zhou, Dayan Wang, Wei Deng, Qi Lv, Yila Ma, Fengdi Li, Huihui Sun, Lingjun Zhan, Hua Zhu, Chunmei Ma, Yuelong Shu, Chuan Qin

**Affiliations:** 1 Institute of Laboratory Animal Sciences, Chinese Academy of Medical Sciences (CAMS) and Comparative Medicine Center, Peking Union Medical College (PUMC), Key Laboratory of Human Disease Comparative Medicine, Ministry of Health, Beijing, China; 2 State Key Laboratory for Molecular Virology and Genetic Engineering, Chinese National Influenza Center, National Institute for Viral Disease Control and Prevention, China CDC, Beijing, China; University of Cambridge, United Kingdom

## Abstract

The novel pandemic A (H1N1) virus was first identified in Mexico in April 2009 and quickly spread worldwide. Like all influenzas, the H1N1 strain-specific properties of replication, virulence, and pathogenicity are a result of the particular genomic sequence and concerted expression of multiple genes. Thus, specific mutations may support increased virulence and may be useful as biomarkers of potential threat to human health. We performed comparative genomic analysis of ten strains of the 2009 pandemic A (H1N1) influenza viruses to determine whether genotypes associated with clinical phenotypes, which ranged from mild to severe illness and up to lethal. Virus replication capacity was tested for each strain *in vitro* using cultured epithelial cells, while virulence and pathogenicity were investigated *in vivo* using the BALB/c mouse model. The results indicated that A/Sichuan/1/2009 strain had significantly higher replication ability and virulence than the other strains, and five unique non-synonymous mutations were identified in important gene-encoding sequences. These mutations led to amino acid substitutions in HA (L32I), PA (A343T), PB1 (K353R and T566A), and PB2 (T471M), and may be critical molecular determinants for replication, virulence, and pathogenicity. Our results suggested that the replication capacity *in vitro* and virulence *in vivo* of the 2009 pandemic A (H1N1) viruses were not associated with the clinical phenotypes. This study offers new insights into the transmission and evolution of the 2009 pandemic A (H1N1) virus.

## Introduction

The first influenza pandemic of the 21^st^ century was declared with the emergence of a novel influenza A (H1N1) strain in Mexico and the United States in April 2009 [1]. Genetic analysis of this novel virus revealed that it is composed of six gene segments which were derived from the triple-reassortant swine lineage and two others from the Eurasian avian-like swine lineage [1]. Thus far, the A (H1N1) influenza has caused a relatively mild pandemic, with a clinical spectrum ranging from slight upper respiratory tract irritation to severe pneumonia leading to acute respiratory distress syndrome [2]. Sporadic cases have occurred in which infection led to death, but those individuals most often had impaired immune status prior to influenza exposure. It is interesting to note that the Spanish influenza pandemic of 1918 and the Hong Kong influenza pandemic of 1968 were both characterized by a first wave of cases which elicited relatively mild illness, followed by a second wave of cases of fulminant disease [3]. The viral molecular mechanisms underlying this robust increase in disease severity have remained elusive; however, it has been hypothesized that pandemic viruses rely on advantageous genetic mutations to adapt to the human host upon zoonotic transmission. As a result, the newly evolved virus will generate a wave of more virulent cases than the first wave. Such genetic adaptation could also occur via gene reassortment events between co-circulating influenza A viruses in the human population.

The virulence, pathogenicity, and host range of influenza viruses have been intensely studied and many diverse factors have been implicated in each. In particular, virus-specific determinants encoded by the viral genome have been defined as principal components of virus survival and pathogenesis; these include the external surface glycoproteins hemagglutinin (HA) and neuraminidase (NA), which interact with host membrane-bound sialic acids [4], [5], [6], [7]. In addition, influenza encodes three polymerase proteins, which have been characterized as important determinants of strains H5N1 and H7N7and necessary for transmission of the 1918 H1N1 virus [5], [6], [8], [9]. The two nonstructural proteins PB1-F2 [10], [11] and NS1 [12] have also been implicated in the virulence capacities of H5N1 and 1918 H1N1 viruses. Intriguingly, genome sequence analysis of the 2009 pandemic A (H1N1) viruses revealed a striking absence of markers associated with high pathogenicity in avian and mammalian species, including the multibasic HA cleavage site [13] and the lysine at position 627 in the PB2 protein [14], [15], [16], [17].

To better understand the potential consequences of viral genetic variations on infection characteristics, we investigated the genomic polymorphisms that occurred among ten strains of the 2009 pandemic A (H1N1) viruses. Virus replication was analyzed in an *in vitro* cell culture system, and virulence and pathogenicity were tested *in vivo* in a mouse model. The findings from our study provide insights into the functional contributions of viral genomic polymorphisms in virus replication, virulence, and pathogenicity, and implicate molecular evolution as a significant driving force behind the 2009 pandemic A (H1N1) influenza virus.

## Materials and Methods

### Viruses

The main background information for all ten virus strains is listed in Table 1. The naming conventions follow the pattern: Type/Geographic Location/Strain Number/Year of Isolation. The A/California/04/2009 and A/California/07/2009 are considered the prototypic strains of the 2009 pandemic A (H1N1) influenza viruses. The A/Sichuan/1/2009 strain was isolated from the first reported case of 2009 pandemic A (H1N1) influenza virus infection in China; the patient was a Chinese student who had returned from the United States in May 2009. The patient reported that no illness symptoms were experienced during the flight from St. Louis to Beijing, but fever developed on the following day during the flight from Beijing to Sichuan. The source of infection remains unknown [18]. A/Sichuan-Wenjiang/SWL456/2009 was collected from a deceased patient, and A/Guangdong/SWL28/2009 was collected from a patient with severe clinical symptoms. A/California/04/2009, A/California/07/2009, A/Sichuan/1/2009, A/Shandong/1/2009, A/Beijing/3/2009, A/Fujian/1/2009, A/Shanghai/1/2009, and A/Jiangsu/1/2009 viruses were all collected from patients with mild symptoms. The passage history of these viruses is listed in Table 1. All viruses were propagated in Madin–Darby canine kidney (MDCK) cells. The 50% tissue culture infectious dose (TCID_50_) was determined by using serial titration of viruses in MDCK cells, and the titers were calculated according to the Reed-Muench method [19].

**Table 1:** The main background information of the ten 2009 pandemic A (H1N1) influenza viruses investigated in this study.

Strain	Patient clinical symptom	Collection date(YYYY/MM/DD)	Collection location	Host(Sex, age in years)	Passage#
A/California/04/2009	Mild	2009/04/01	California, USA	Male, 10	E4
A/California/07/2009	Mild	2009/04/09	California, USA	Male, 54	E4
A/Sichuan/1/2009	Mild	2009/05/09	Sichuan, China	Male, 30	E3
A/Shandong/1/2009	Mild	2009/05/10	Shandong, China	Male, 19	E4
A/Beijing/3/2009	Mild	2009/05/20	Beijing, China	Male, 19	C2
A/Fujian/1/2009	Mild	2009/05/22	Fujian, China	Female, 1	E2
A/Shanghai/1/2009	Mild	2009/05/23	Shanghai, China	Male, 30	E2
A/Jiangsu/1/2009	Mild	2009/06/15	Jiangsu, China	Male, 46	C1
A/Guangdong/SWL28/2009	Severe	2009/08/08	Guangdong, China	Male, 17	C1
A/Sichuan-Wenjiang/SWL456/2009	Dead	2009/11/06	Sichuan, China	Male, 53	C2

# : E, propagated in in the allantoic cavities of chicken embryonated eggs; C, propagated in MDCK cells.

### Cell infection

MDCK cells were maintained in Dulbecco's modified Eagle's medium (DMEM) (Invitrogen) supplemented with 10% fetal bovine serum. Viruses (10^2^ TCID_50_) were added to respective cell monolayers in 35-mm dishes (Corning). After 60 min adsorption at 37°C, cells were washed and fed with minimum essential medium containing tosylsulfonyl phenylalanyl chloromethyl ketone (TPCK)-treated trypsin (0.5 µg/ml) (Sigma) and antibiotics (Sigma). This time point was designated as 0 hour post-infection (h.p.i.). Viral supernatants (100 µl) were harvested at 12, 24, 36, 48, 56, 72, 96 h.p.i. and separated from cell debris by centrifugation at 3000×*g* for 10 min.

### Mice challenge

Female 5-week-old specific pathogen-free BALB/c mice used in this study were obtained from the Institute of Laboratory Animal Sciences, Beijing, China. Mice were anesthetized and inoculated intranasally with virus (n = 23 per group; 50 µl of 10^6^ TCID_50_). In each group 10 mice were chosen at random for daily monitoring for signs of disease and mortality, up to 14 days post inoculation (d.p.i.). Ten of the remaining mice in each group were euthanized at 5 d.p.i. to obtain lung tissue biopsies for use in subsequent quantification of viral nucleotide material and pathological investigations. The final 3 mice were sacrificed at 5 d.p.i. and lungs were collected for viral titer detection. All procedures were approved by the Institute of Animal Use and Care Committee of the Institute of Laboratory Animal Science, Peking Union Medical College (approval number ILAS-PC-2010-002).

### Plaque assay

The infectivity of viruses released from infected MDCK cells into supernatants and in the homogenized lung tissues of challenged mice were determined by plaque assay and expressed as log_10_ plaque-forming units (PFU) per milliliter [20]. Briefly, confluent MDCK cells were incubated at 37°C for 1 hour with 10-fold serial dilutions of virus. The cells were then washed and overlaid with minimum essential medium containing 0.3% bovine serum albumin (BSA), 0.9% Bacto agar, and 1 µg/ml TPCK-treated trypsin and antibiotics. Plaques were visualized by neutral red staining and counted after 48 hours of incubation at 37°C.

### Real-time PCR

Total RNA was isolated from homogenized lung tissues by using the RNeasy Mini Kit (Qiagen), according to the manufacturer's instructions. RNA was resuspended in 30 µl nuclease-free water and stored at −80°C. First-strand cDNA was synthesized from RNA (8 µl) by random primers in a SuperScript II reverse transcriptase (200 U) reaction mixture (20 µl) (Invitrogen). SYBR Green real-time quantitative PCR was performed with a StepOne PCR system (ABI) using cDNA (2 µl) and a reaction mixture (20 µl) containing 2× SYBR Green PCR Master Mix (10 µl) (ABI), 10 µM forward and reverse primers (1 µl each: SW-HA F786, 5′-AATAACATTAGAAGCAACTGG-3′; SW-HA R920, 5′-AGGCTGGTGTTTATRGCACC-3′), and nuclease-free water. The following thermal cycling conditions were used: 94°C for 3 min, followed by 35 cycles of 94°C for 30 s, 58°C for 30 s, and 72°C for 30 s.

### Pathological analysis

Immediately following euthanasia, mouse lungs were removed, inflated, and fixed with 10% neutral buffered formalin overnight at 4°C. Subsequently, the formalin-preserved lung samples were embedded in paraffin and sectioned. Serial 4-µm sections were stained with Hematoxylin and Eosin (H&E) and examined for pathological changes that corresponded to infection. Images were obtained on an Olympus BX-50 light microscope at 40× magnification.

### Receptor-binding assay

Synthetic SA-α-2,3-lactose(3′SL-PAA)-Biotin, 3′SL-DI-PAA-Biotin, SA-α-2,6-N-acetyl lactosamine(6′SLN-PAA)-Biotin, 6′SLN-DI-PAA-Biotin were provided by The Scripps Research Institute (La Jolla, CA, USA). The receptor binding procedure was carried out as described elsewhere, with some modifications [21]. Briefly, 96-well flat–bottom polystyrene plates were coated with serial dilutions of sialyglycopolymers, and 32 HAU of live virus were added per well. Rabbit antisera against the A/California/07/2009 strain were diluted in phosphate buffered saline containing 1% bovine serum albumin and then added to each well. Bound antibody was detected by sequential additions of HRP-conjugated anti-rabbit IgG antibody and tetramethylbenzidine substrate solution and reading the spectrophotometric absorbance at 450 nm. Each sample was determined in triplicate.

### DNA sequencing and analysis

All gene segments for each of the ten viruses were amplified by high fidelity PCR (KOD-Plus DNA polymerase; Toyobo, Japan). The PCR products were purified and sequenced (Invitrogen). Sequences of each gene of A/Guangdong/SWL28/2009 and A/Sichuan-Wenjiang/SWL456/2009 viruses were deposited in GenBank. Sequences of all viruses were analyzed and aligned using ClustalW software (version 1.83).

### Statistical analysis

Statistical analysis of the viral load and titer were performed using SPSS 11.5 software. The Duncan and least significant differences methods were used for comparisons among multiple groups by one-way ANOVA. The Kaplan-Meier method was used to estimate the probability of survival of infected mice. A probability value of 0.05 was considered as statistically significant.

## Results

### *In vitro* comparison of virus replication in cells

**Figure 1:**
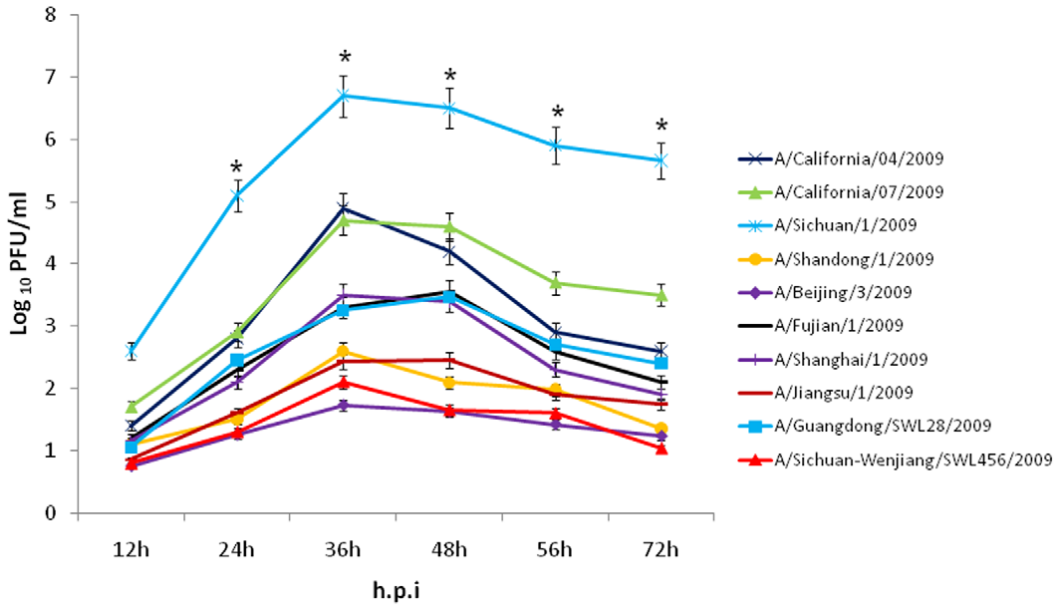
Replication kinetics of the ten 2009 pandemic A (H1N1) influenza viruses in MDCK cells. Each data point represents the mean viral yield (log_10_ PFU/ml) from three individually infected wells ± SD. *P<0.05 compared to the values of other viruses (one-way ANOVA).

Analysis of virus replication kinetics in MDCK cells revealed that all ten viruses reached their peak titers at 36 to 48 h.p.i, and the titers of A/Sichuan/1/2009 were significantly higher (*P*<0.05) than those obtained with other strains after 12 h.p.i (Fig. 1).

### *In vivo* comparison of virulence in the mice model

**Figure 2:**
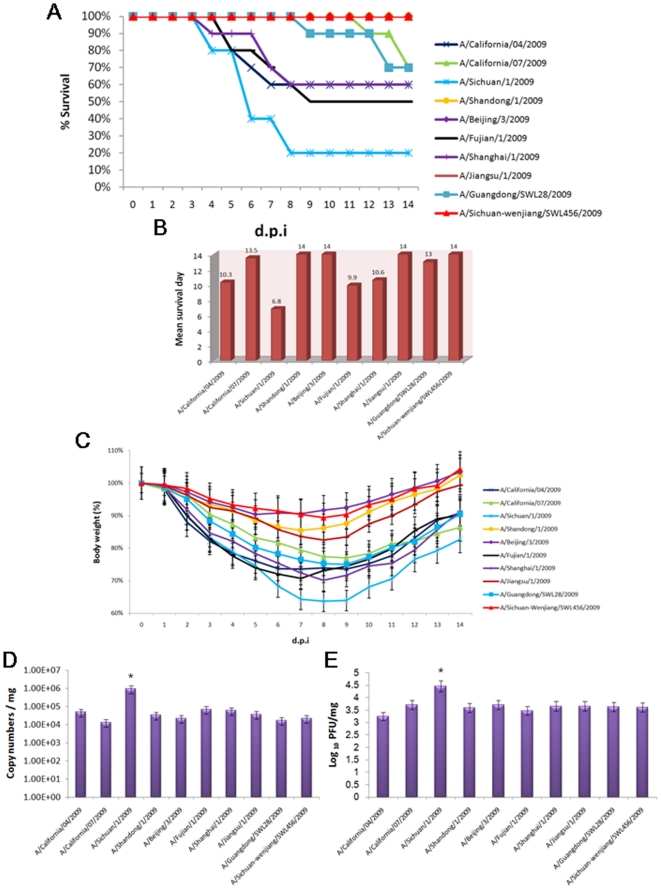
Virulence comparison of the ten 2009 pandemic A (H1N1) influenza viruses in BALB/c mice. Mice were anesthetized and inoculated intranasally with virus (n = 23 per group; 50 µl of 10^6^ TCID_50_). Ten randomly selected mice were monitored daily for signs of disease and mortality, up to 14 d.p.i. for (A), (B), and (C) research, whereas ten of the remaining mice from each group were euthanized at 5 d.p.i. to obtain lung tissue biopsies for use in (D). The final three mice were also sacrificed at 5 d.p.i. and their lungs were collected for (E) detection. (A) Survival percentage of mice. (B) Mean survival days for each challenged group. (C) Body weight changes of infected mice. Mean body weight and SD were calculated as percentage of body weight and compared to those at 0 d.p.i. (D) Viral RNA loads in lung tissues at 5 d.p.i. Data are presented as mean viral loads per microgram ± SD. (E) Viral titers in lung tissues at 5 d.p.i. Data are presented as mean log_10_ PFU/mg. * *P*<0.05 compared to the values of other viruses (one-way ANOVA).

To compare the virulence of the ten pandemic A (H1N1) influenza viruses *in vivo*, we intranasally challenged BALB/c mice with each virus and evaluated mortality, mean survival days, total body weight loss, viral RNA loads and titers in lung tissues. The percentages of mice that survived the infections are shown in Figure 2A. Eighty percent of mice inoculated with the A/Sichuan/1/2009 virus died by 14 d.p.i. Mice infected with the A/California/04/2009 and A/California/07/2009 had survival ratios of 60% and 70%, respectively. Furthermore, the A/Fujian/1/2009, A/Shanghai/1/2009, and A/Guangdong/SWL28/2009 viruses were associated with survival ratios ranging from 50–70%. Challenge with A/Shandong/1/2009, A/Beijing/3/2009, A/Jiangsu/1/2009, or A/Sichuan-Wenjiang/SWL456/2009 was associated with 100% survival. The mean survival days [22] of each mice group are shown in Figure 2B. The A/Sichuan/1/2009 virus challenged mice survived only 6.8 days, and represented the shortest survival time of all groups. Meanwhile, challenge with A/Sichuan/1/2009 led to nearly 40% body weight loss by 7–9 d.p.i, after which the mice began to steadily regain the lost weight over the course of the remaining observation period. The body weight losses of mice which were challenged with A/California/04/2009, A/California/07/2009, A/Fujian/1/2009, A/Shanghai/1/2009, or A/Guangdong/SWL28/2009 ranged from 20% to 30%, whereas those inoculated with A/Beijing/3/2009, A/Shandong/1/2009, A/Jiangsu/1/2009, or A/Sichuan-Wenjiang/SWL456/2009 were less than 20% (Fig. 2C).

To further investigate the differences in pathogenicity of each virus, we determined the degree of virus replication occurring in lung tissues of challenged mice. The mean viral RNA loads (copy numbers per mg ± standard deviation (SD); Fig. 2D) and viral titers (log_10_ PFU per mg; Fig. 2E) were determined at day 5 post-infection, when virus shedding is known to reach its peak according to our previous studies [23], [24]. Mice inoculated with the A/Sichuan/1/2009 virus presented with the highest RNA copy numbers of virus in the lungs and significantly higher (*P*<0.05) viral RNA load than the other nine viruses. Consistent with the viral RNA load result, lung tissues of mice challenged with A/Sichuan/1/2009 exhibited the highest viral titer.

**Figure 3:**
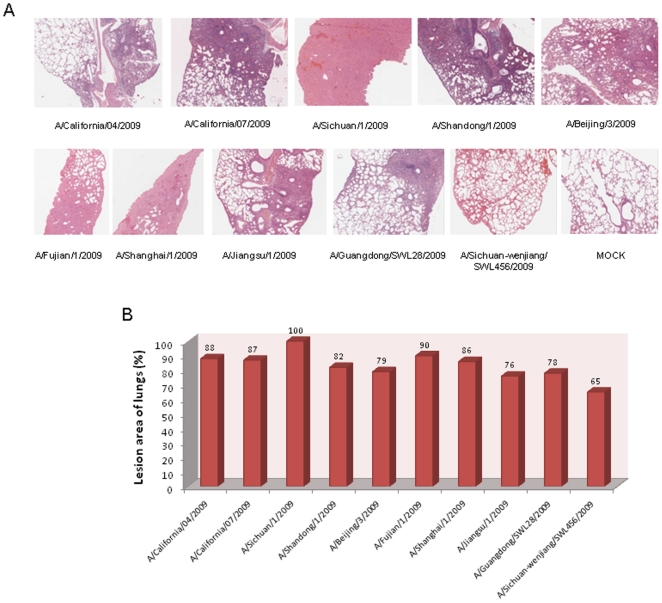
Pathological analysis of the lung tissues of challenged BALB/c mice. Ten mice from each group were euthanized at 5 d.p.i. to obtain lung tissue biopsies, and for each lung three 4-µm sections were stained with H&E for pathological investigations. (A) Representative sections of H&E stained lung tissues from 10^6^ TCID_50_ intranasally challenged mice. (B) Percentage of lesion area in lung tissues.

In order to examine the differential pathological changes that may take place in the lungs of mice challenged with the different strains of virus, lung tissues were isolated day 5 post-infection. All viruses were found to have replicated efficiently in the lung tissues. In addition, all lung tissue samples exhibited characteristic pathology of influenza infection, including inflammatory hyperaemia, hemorrhage, edema, and exudative pathological changes. Mice lungs harboring A/Sichuan/1/2009 virus exhibited the most robust pathophysiology, with lesions occurring in 100% of the lung tissue sections examined. Mice inoculated with A/California/04/2009, A/California/07/2009, A/Shanghai/1/2009, and A/Fujian/1/2009 viruses exhibited pathological changes in >85% of the lung tissue sections. Infection with A/Shandong/1/2009, A/Beijing/3/2009, A/Jiangsu/1/2009, A/Guangdong/SWL28/2009, and A/Sichuan-Wenjiang/SWL456/2009 viruses were associated with lesion occurrence in 60–85% of lung tissues (Fig. 3A&B).

### Sequence alignment of A (H1N1) viral genomes

The full-length sequences of all ten viral genomes were obtained by high fidelity PCR and sequenced as described in the Methods section. The sequences of the HA, NA, M2&M1, NEP&NS1, NP, PA, PB1, and PB2 genes in A/Guangdong/SWL28/2009 and A/Sichuan-Wenjiang/SWL456/2009 viruses were deposited in GenBank under accession numbers HM051340 to HM051347, and HM051348 to HM051355, respectively. Genomic polymorphisms found in each of the ten viruses are listed in Table 2. In particular, unique non-synonymous mutations were found in some of the virulence genes of A/Sichuan/1/2009: a Leu-to-Ile variation at position 32 in the HA coding sequence and another four substitutions in the polymerases: Ala-to-Thr at position 343 in PA, Lys-to-Arg at position 353 and Thr-to-Ala at position 566 in PB1, and Thr-to-Met at position 471 in PB2.

**Table 2:** Protein encoding sequences alignment of the ten 2009 pandemic A (H1N1) influenza viruses.

Strain	HA											NA				NEP&NS1		
	32	83	188	189	196	197	203	293	321	402	411	106	248	329	351	25	123	183
	Leu→Ile	Pro→Ser	Gln→Arg	Gln→His	Asp→Asn	Ala→Thr	Ser→Thr	Gln→His	Val→Ile	Lys→Thr	Val→Ile	Val→Ile	Asn→Asp	Asn→Ile	Phe→Tyr	Asn→Asp	Ile→Val	Gly→Arg
A/California/04/2009	C	C	A	A	G	A	T	G	A	A	G	G	A	A	T	A	A	G
A/California/07/2009	C	C	A	A	G	G	T	G	A	A	G	G	A	A	A	A	A	G
A/Sichuan/1/2009	**A**	T	A	A	G	G	T	G	G	A	G	G	A	A	T	A	A	G
A/Shandong/1/2009	C	T	A	A	G	G	T	T	G	C	G	A	G	A	T	A	A	G
A/Beijing/3/2009	C	T	A	A	G	G	T	G	G	A	G	A	G	A	T	A	A	G
A/Fujian/1/2009	C	T	A	A	G	G	A	G	G	A	G	A	G	A	T	A	G	G
A/Shanghai/1/2009	C	T	A	A	G	G	A	G	G	A	A	A	G	A	T	A	G	A
A/Jiangsu/1/2009	C	T	A	A	G	G	T	G	G	A	G	G	A	T	T	G	A	G
A/Guangdong/SWL28/2009	C	T	G	T	A	G	A	G	G	A	G	A	G	A	T	A	G	G
A/Sichuan-Wenjiang/SWL456/2009	C	T	A	A	G	G	A	G	G	A	G	A	G	A	T	A	G	G

Strain	M1	NP		PA							PB1				PB2			
	94	100	373	14	222	224	343	343	557	581	353	566	587	735	89	471	480	526
	Asp→Asn	Val→Ile	Thr→Ile	Val→Ile	Asn→Lys	Ser→Pro	Ala→Thr	Ala→Gly	Val→Met	Met→Leu	Lys→Arg	Thr→Ala	Val→Ala	Ile→Val	Val→Met	Thr→Met	Val→Ile	Lys→Arg
A/California/04/2009	G	G	C	G	C	C	G	C	G	A	A	A	T	A	G	C	G	A
A/California/07/2009	G	G	C	G	C	C	G	C	G	A	A	A	T	A	G	C	G	A
A/Sichuan/1/2009	G	G	C	G	C	T	**A**	C	G	A	**G**	**G**	T	A	G	**T**	G	A
A/Shandong/1/2009	G	A	C	G	C	T	G	C	A	A	A	A	T	G	G	C	A	A
A/Beijing/3/2009	A	A	C	G	C	T	G	C	G	A	A	A	T	A	G	C	G	A
A/Fujian/1/2009	G	A	C	G	C	T	G	C	G	A	A	A	T	A	G	C	G	A
A/Shanghai/1/2009	G	A	C	A	C	T	G	C	G	A	A	A	T	A	G	C	G	A
A/Jiangsu/1/2009	G	G	T	G	C	T	G	G	G	C	A	A	C	A	G	C	G	G
A/Guangdong/SWL28/2009	G	A	C	G	A	T	G	C	G	A	A	A	T	A	G	C	G	A
A/Sichuan-Wenjiang/SWL456/2009	G	A	C	G	C	T	G	C	G	A	A	A	T	A	A	C	G	A

The numbering of the residues are from the first amino acid in the methionine start site of each gene of influenza viruses.

### Effect of HA Leu32Ile substitution on receptor binding ability

**Figure 4:**
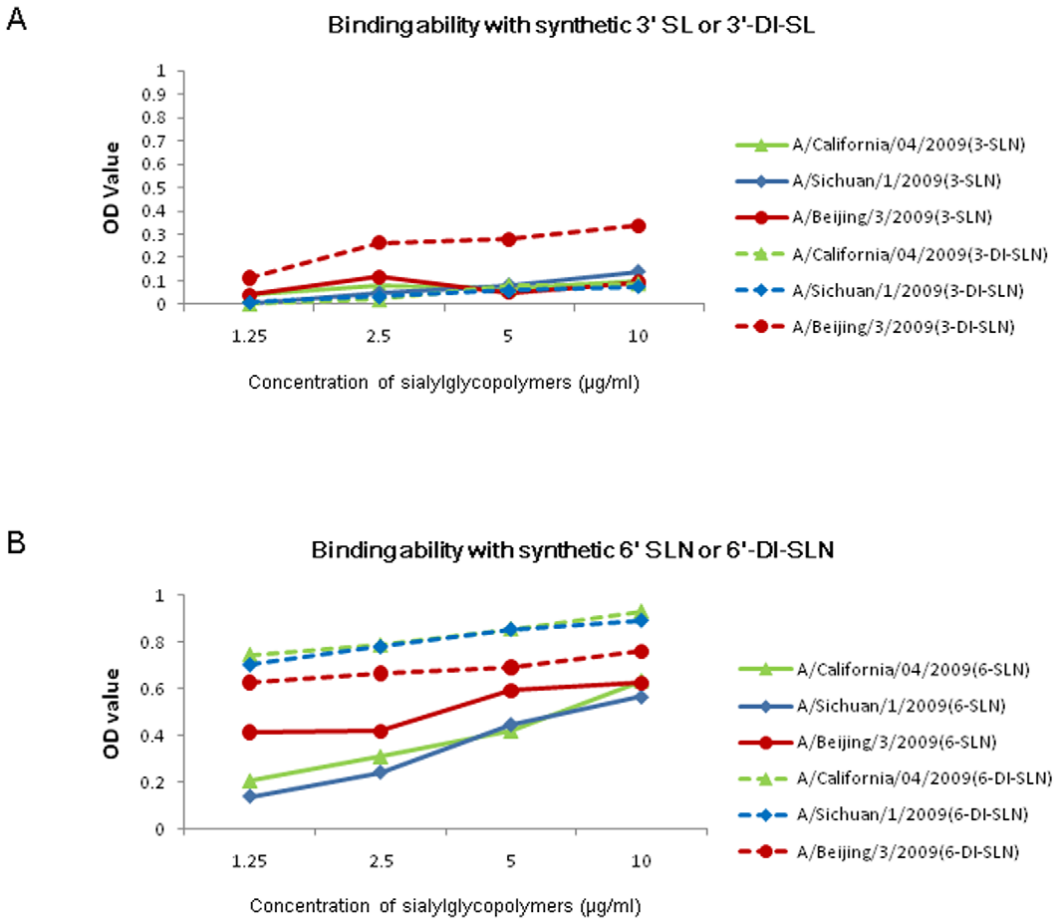
Direct binding assay with synthetic sialylglycopolymers. (A) Affinity to synthetic 3′SL or 3′DI-SL. (B) Affinity to synthetic 6′SLN or 6′DI-SLN.

Previous studies have suggested that HA mutations may act to increase virus virulence by affecting the affinity of HA binding to host sialyl receptors. We examined three representative strains (A/California/04/2009, A/Sichuan/1/2009, A/Beijing/3/2009) and measured their HA receptor binding abilities to synthetic sialic substrates. No significant difference was observed in the affinities of the three HAs for binding to human-type 6′SLN or 6′DI-SLN and avian-type 3′SL or 3′DI-SL receptors (*P*>0.05) (Fig. 4). This finding demonstrated that the HA Leu32Ile mutation in A/Sichuan/1/2009 contributed minimally, or not at all, to the uniquely high virulence phenotype.

## Discussion

Influenza viruses cause epidemics and pandemics through antigenic drift or antigenic shift [25]. The 2009 pandemic A (H1N1) virus has generally been associated with mild disease and a relatively low mortality rate; however, sporadic severe or fatal cases have been reported. Certainly, discrepancies in individual immunity may support such diverse virulence of the 2009 pandemic A (H1N1) viruses, but it is as likely that some yet undefined viral molecular mechanisms are at play [3], [26]. Mutations in specific regions of a given viral genome are known to result in increased virulence, and may lead to a more severe pandemic. In this study, we investigated the molecular basis of the 2009 pandemic A (H1N1) virus that mediates its uniquely robust characteristics of replication, virulence, and pathogenicity.

We first examined the viral replication kinetics of all ten viruses by using an *in vitro* infection model of MDCK cells. The A/Sichuan/1/2009 strain had an obvious enhanced replication ability, which led to increased peak titers of roughly 3–4 orders of magnitude higher than the other viruses, with the exception of A/California/04/2009 and A/California/07/2009 strains. These two viruses exhibited 2–3 orders of magnitude lower viral titers, as compared with A/Sichuan/1/2009 after 12 h.p.i. We also performed *in vivo* analysis using the BALB/c mouse model. Pronounced virulence and pathogenicity of the A/Sichuan/1/2009 virus were both observed; mice infected with this virus had the lowest survival ratio, shortest mean survival days, most extensive body weight loss, highest virus RNA copy numbers and titers in lung tissues, most severe pathological changes, and largest lesion areas in lung tissues. Taken together, these *in vitro* and *in vivo* results indicated that the A/Sichuan/1/2009 virus has the strongest replication ability in MDCK cells and virulence in the BALB/c mouse model.

Sequence alignment among the ten A (H1N1) viruses revealed that the A/Sichuan/1/2009 virus genome harbors five unique mutations: L32I in HA; A343T in PA; K353R and T566A in PB1; and T471M in PB2. Receptor binding assay showed that despite having a non-synonymous mutation in the HA gene, the A/Sichuan/1/2009 virus did not have increased binding affinity for synthetic sialyl receptors, including the human type 6′SLN and the avian type 3′SL. Thus, it is unlikely that the HA Leu32Ile mutation acts as a key molecular determinant for the increased virulence of A/Sichuan/1/2009. While previous studies have associated another mutation in the HA receptor binding domain, Asp222Gly, with enhanced virulence [24], [27], [28], [29], [30], [31], it remains to be confirmed whether HA affinity for sialyl receptors is directly responsible for the enhanced virulence of the 2009 pandemic A (H1N1) influenza viruses. However, Glinsky and Melidou et al. presented findings to suggest that the HA Gln293His amino acid change may be associated with increased disease severity [32], [33].

The polymerase genes of influenza viruses are considered to be extremely important for virulence. Codon position 515 in the PA protein, for example, is significantly related to pathogenicity of an H5N1 virus in ducks [34]. A reverse genetics study demonstrated that mutations in the PB2 protein, Glu627Lys and Asp701Asn, were responsible for virulence in mammalian species [8]. The amino acid at position 701 in PB2 has been characterized as crucial to replication and lethality of duck-originated H5N1 viruses in mice [35]. The same PB2 amino acid residue was shown to contribute to increased lethality of an H7N1 avian influenza virus in a mouse model [36]. However, Jagger et al. reported that influenza viruses containing mutations at residues 627 and 701 in the context of the pandemic A (H1N1) virus polymerase complex have attenuated virulence both in cell culture and the mouse model system [17]. Recently, the PB2 mutation Thr271Ala was found to enhance polymerase activity and viral growth in human cells [37]. PB2 residue 158 has been described as a pathogenic determinant of the pandemic H1N1 and H5 influenza A viruses in mice [38]. Moreover, PB1-F2, a short viral protein of approximately 90 amino acids expressed from a +1 reading frame in the PB1 gene segment, was also reported as another virulence determinant; the serine at position 66 in PB1-F2 was associated with increased disease pathology in a mouse model [11], [39], [40]. Interestingly, the 2009 pandemic A (H1N1) influenza viruses do not encode a PB1-F2 protein, due to the presence of three stop codons in the gene sequence. However, Wanitchang et al. and Hai et al. reported that reinstatement of PB1-F2 elicited a minimal effect on virulence of the pandemic A (H1N1) virus in various mammalian models [41], [42].

All the above data suggest that the polymerase complex mediates virulence of influenza viruses. Indeed, the four residues mutated within the polymerase complex of A/Sichuan/1/2009 virus may indicate key virulence determinants of the 2009 pandemic A (H1N1) viruses. However, it is still uncertain whether the increased virulence is caused by any single substitution or some combinations of the four. Further investigations using reverse genetics are likely to provide insights into this question.

Epidemiologic analysis showed that the ten viruses examined in this study could be divided into three groups based on time and region of prevalence. Group I contained the A/California/04/2009 and A/California/07/2009 viruses, which represented the very first 2009 pandemic A (H1N1) influenza patients reported. Group II contained the A/Sichuan/1/2009 strain, which was isolated from the first reported case in China. As these Group II strains were isolated during the earliest phase of the Chinese pandemic, this group also represented the period of H1N1 global dissemination [43]. Group III contained the other seven strains, which were representative of domestic infections and were all collected in China. The same HA sequence polymorphism found in A/Sichuan/1/2009 existed in other 2009 pandemic A (H1N1) strains which were collected across the globe from April to June of 2009. Most of these other strains represented the first waves of H1N1 in their respective countries, including Canada, Mexico, Nicaragua, France, Finland, and the United States. Interestingly, PA mutation in A/Sichuan/1/2009 has never been reported before. In contrast, sequence polymorphisms in PB1 and PB2 have been identified in strains collected from the first waves in Canada, Nicaragua, and some cities of the United States, but little information is available about the virulence and pathogenicity of those particular strains.

Belser et al. also used a mouse model to evaluate the virulence of a collection of 2009 A (H1N1) viruses. Their studies ultimately demonstrated that the viruses exhibited mild to moderate virulence in mice. They also performed sequence analysis of their isolates, and identified similar polymorphisms to those reported here; molecular features which are frequently found among viruses of high pathogenicity in mammalian models were not detected in either their or our studies [44]. Although it was reported that hypercytokinemia is not a general feature of infection with the 2009 pandemic A (H1N1) viruses, including those isolated from fatal cases [44], however, it is likely that some, at least subtle, affects from the cytokine storm exist to the virulence [45], [46], [47], [48], [49], [50]. Since none of the genetic changes identified in A/Sichuan/1/2009 involve critical domain/motifs in HA or in the polymerase complex [8], [51], [52], [53], further study addressing the potential for host-based differences in immunologic response is warranted.

Our results also suggested that the virulence of the 2009 pandemic A (H1N1) viruses were not associated with the clinical phenotypes of the corresponding patients; A/Sichuan-Wenjiang/SWL456/2009 and A/Guangdong/SWL28/2009, viruses collected from a deceased and severely ill patient respectively, showed much lower replication ability, virulence, and pathogenicity than A/Sichuan/1/2009, a virus from a patient with mild symptoms. Previous investigations into a series of severe and fatal cases of the pandemic H1N1 influenza revealed that pregnancy, obesity, diabetes, and other co-morbid conditions were associated with severe disease [54], [55]. Therefore, it is reasonable to hypothesize that the two patients with A/Sichuan-Wenjiang/SWL456/2009 or A/Guangdong/SWL28/2009 virus infection were suffering from concomitant complicating diseases. Unfortunately, time and anonymity concerns have made it impossible to now trace the clinical features and anamnesis of these two patients. It is interesting to note, here, that Belser et al. deduced the same conclusion, citing that 2009 H1N1 viruses isolated from fatal cases did not demonstrate enhanced virulence in a mouse model as compared with isolates from mild human cases [44].

In summary, we found that the genomic polymorphisms that characterize each of the 2009 pandemic A (H1N1) influenza viruses contribute to virus replication ability, virulence, and pathogenicity. Non-synonymous polymorphisms associated with amino acid substitutions in PA (A343T), PB1 (K353R and T566A), and PB2 (T471M) were identified as potential key virulence determinants. Meanwhile, the replication ability *in vitro* and virulence *in vivo* of the 2009 pandemic A (H1N1) viruses were not associated with the clinical phenotypes exhibited by the corresponding patients. These preliminary observations contribute to our understanding of the genetic process of transmission and evolution of the 2009 pandemic A (H1N1) influenza viruses. Because of the functional importance of the polymerase complex in replication, virulence, and pathogenicity, the mutations described here deserve further investigation and may lead to discoveries of new vaccines and therapeutic drugs against this globally important virus.

## References

[1] Garten RJ, Davis CT, Russell CA, Shu B, Lindstrom S (2009). Antigenic and genetic characteristics of swine-origin 2009 A(H1N1) influenza viruses circulating in humans.. Science.

[2] Perez-Padilla R, de la Rosa-Zamboni D, Ponce de Leon S, Hernandez M, Quinones-Falconi F (2009). Pneumonia and respiratory failure from swine-origin influenza A (H1N1) in Mexico.. N Engl J Med.

[3] Taubenberger JK (2006). The origin and virulence of the 1918 “Spanish” influenza virus.. Proc Am Philos Soc.

[4] Glaser L, Stevens J, Zamarin D, Wilson IA, Garcia-Sastre A (2005). A single amino acid substitution in 1918 influenza virus hemagglutinin changes receptor binding specificity.. J Virol.

[5] Chen H, Bright RA, Subbarao K, Smith C, Cox NJ (2007). Polygenic virulence factors involved in pathogenesis of 1997 Hong Kong H5N1 influenza viruses in mice.. Virus Res.

[6] Tumpey TM, Basler CF, Aguilar PV, Zeng H, Solorzano A (2005). Characterization of the reconstructed 1918 Spanish influenza pandemic virus.. Science.

[7] Tumpey TM, Garcia-Sastre A, Taubenberger JK, Palese P, Swayne DE (2004). Pathogenicity and immunogenicity of influenza viruses with genes from the 1918 pandemic virus.. Proc Natl Acad Sci U S A.

[8] Hatta M, Gao P, Halfmann P, Kawaoka Y (2001). Molecular basis for high virulence of Hong Kong H5N1 influenza A viruses.. Science.

[9] Hatta M, Hatta Y, Kim JH, Watanabe S, Shinya K (2007). Growth of H5N1 influenza A viruses in the upper respiratory tracts of mice.. PLoS Pathog.

[10] Zamarin D, Ortigoza MB, Palese P (2006). Influenza A virus PB1-F2 protein contributes to viral pathogenesis in mice.. J Virol.

[11] Conenello GM, Zamarin D, Perrone LA, Tumpey T, Palese P (2007). A single mutation in the PB1-F2 of H5N1 (HK/97) and 1918 influenza A viruses contributes to increased virulence.. PLoS Pathog.

[12] Geiss GK, Salvatore M, Tumpey TM, Carter VS, Wang X (2002). Cellular transcriptional profiling in influenza A virus-infected lung epithelial cells: the role of the nonstructural NS1 protein in the evasion of the host innate defense and its potential contribution to pandemic influenza.. Proc Natl Acad Sci U S A.

[13] Webster RG, Bean WJ, Gorman OT, Chambers TM, Kawaoka Y (1992). Evolution and ecology of influenza A viruses.. Microbiol Rev.

[14] Subbarao EK, London W, Murphy BR (1993). A single amino acid in the PB2 gene of influenza A virus is a determinant of host range.. J Virol.

[15] Zhu H, Wang J, Wang P, Song W, Zheng Z (2010). Substitution of lysine at 627 position in PB2 protein does not change virulence of the 2009 pandemic H1N1 virus in mice.. Virology.

[16] Herfst S, Chutinimitkul S, Ye J, de Wit E, Munster VJ (2010). Introduction of virulence markers in PB2 of pandemic swine-origin influenza virus does not result in enhanced virulence or transmission.. J Virol.

[17] Jagger BW, Memoli MJ, Sheng ZM, Qi L, Hrabal RJ (2010). The PB2-E627K mutation attenuates viruses containing the 2009 H1N1 influenza pandemic polymerase.. MBio.

[18] Cao B, Li XW, Mao Y, Wang J, Lu HZ (2009). Clinical features of the initial cases of 2009 pandemic influenza A (H1N1) virus infection in China.. N Engl J Med.

[19] Reed LJ, Muench H (1938). A simple method of estimating fifty percent endpoints.. The American Journal of Hygiene.

[20] Hayden FG, Cote KM, Douglas RG (1980). Plaque inhibition assay for drug susceptibility testing of influenza viruses.. Antimicrob Agents Chemother.

[21] Auewarakul P, Suptawiwat O, Kongchanagul A, Sangma C, Suzuki Y (2007). An avian influenza H5N1 virus that binds to a human-type receptor.. J Virol.

[22] Davies WL, Grunert RR, Haff RF, McGahen JW, Neumayer EM (1964). Antiviral Activity of 1-Adamantanamine (Amantadine).. Science.

[23] Bao L, Xu L, Zhan L, Deng W, Zhu H (2010). Challenge and polymorphism analysis of the novel A (H1N1) influenza virus to normal animals.. Virus Res.

[24] Xu L, Bao L, Lv Q, Deng W, Ma Y (2010). A single-amino-acid substitution in the HA protein changes the replication and pathogenicity of the 2009 pandemic A (H1N1) influenza viruses in vitro and in vivo.. Virol J.

[25] Cheung TK, Poon LL (2007). Biology of influenza a virus.. Ann N Y Acad Sci.

[26] Kash JC, Tumpey TM, Proll SC, Carter V, Perwitasari O (2006). Genomic analysis of increased host immune and cell death responses induced by 1918 influenza virus.. Nature.

[27] Potdar VA, Chadha MS, Jadhav SM, Mullick J, Cherian SS (2010). Genetic characterization of the influenza A pandemic (H1N1) 2009 virus isolates from India.. PLoS One.

[28] Ikonen N, Haanpaa M, Ronkko E, Lyytikainen O, Kuusi M (2010). Genetic diversity of the 2009 pandemic influenza A(H1N1) viruses in Finland.. PLoS One.

[29] Kilander ARR, Dudman S, Hungnes O (2010). Observed association between the HA1 mutation D222G in the 2009 pandemic influenza A(H1N1) virus and severe clinical outcome, Norway 2009–2010.. Euro Surveill.

[30] Liu Y, Childs RA, Matrosovich T, Wharton S, Palma AS (2010). Altered receptor specificity and cell tropism of D222G hemagglutinin mutants isolated from fatal cases of pandemic A(H1N1) 2009 influenza virus.. J Virol.

[31] Mak GC, Au KW, Tai LS, Chuang KC, Cheng KC (2010). Association of D222G substitution in haemagglutinin of 2009 pandemic influenza A (H1N1) with severe disease.. Euro Surveill.

[32] Glinsky GV (2010). Genomic analysis of pandemic (H1N1) 2009 reveals association of increasing disease severity with emergence of novel hemagglutinin mutations.. Cell Cycle.

[33] Melidou A, Gioula G, Exindari M, Chatzidimitriou D, Diza E (2010). Molecular and phylogenetic analysis of the haemagglutinin gene of pandemic influenza H1N1 2009 viruses associated with severe and fatal infections.. Virus Res.

[34] Hulse-Post DJ, Franks J, Boyd K, Salomon R, Hoffmann E (2007). Molecular changes in the polymerase genes (PA and PB1) associated with high pathogenicity of H5N1 influenza virus in mallard ducks.. J Virol.

[35] Li Z, Chen H, Jiao P, Deng G, Tian G (2005). Molecular basis of replication of duck H5N1 influenza viruses in a mammalian mouse model.. J Virol.

[36] Gabriel G, Dauber B, Wolff T, Planz O, Klenk HD (2005). The viral polymerase mediates adaptation of an avian influenza virus to a mammalian host.. Proc Natl Acad Sci U S A.

[37] Bussey KA, Bousse TL, Desmet EA, Kim B, Takimoto T (2010). PB2 residue 271 plays a key role in enhanced polymerase activity of influenza A viruses in mammalian host cells.. J Virol.

[38] Zhou B, Li Y, Halpin R, Hine E, Spiro DJ (2011). PB2 Residue 158 Is a Pathogenic Determinant of Pandemic H1N1 and H5 Influenza A Viruses in Mice.. J Virol.

[39] McAuley JL, Hornung F, Boyd KL, Smith AM, McKeon R (2007). Expression of the 1918 influenza A virus PB1-F2 enhances the pathogenesis of viral and secondary bacterial pneumonia.. Cell Host Microbe.

[40] Conenello GM, Palese P (2007). Influenza A virus PB1-F2: a small protein with a big punch.. Cell Host Microbe.

[41] Wanitchang A, Kramyu J, Jongkaewwattana A (2010). Enhancement of reverse genetics-derived swine-origin H1N1 influenza virus seed vaccine growth by inclusion of indigenous polymerase PB1 protein.. Virus Res.

[42] Hai R, Schmolke M, Varga ZT, Manicassamy B, Wang TT (2010). PB1-F2 expression by the 2009 pandemic H1N1 influenza virus has minimal impact on virulence in animal models.. J Virol.

[43] Nelson M, Spiro D, Wentworth D, Beck E, Fan J (2009). The early diversification of influenza A/H1N1pdm.. PLoS Curr.

[44] Belser JA, Wadford DA, Pappas C, Gustin KM, Maines TR (2010). Pathogenesis of pandemic influenza A (H1N1) and triple-reassortant swine influenza A (H1) viruses in mice.. J Virol.

[45] Seo SH, Hoffmann E, Webster RG (2002). Lethal H5N1 influenza viruses escape host anti-viral cytokine responses.. Nat Med.

[46] Safronetz D, Rockx B, Feldmann F, Belisle SE, Palermo RE (2011). Pandemic swine-origin H1N1 influenza A virus isolates show heterogeneous virulence in macaques.. J Virol.

[47] Bermejo-Martin JF, Ortiz de Lejarazu R, Pumarola T, Rello J, Almansa R (2009). Th1 and Th17 hypercytokinemia as early host response signature in severe pandemic influenza.. Crit Care.

[48] de Jong MD, Simmons CP, Thanh TT, Hien VM, Smith GJ (2006). Fatal outcome of human influenza A (H5N1) is associated with high viral load and hypercytokinemia.. Nat Med.

[49] Skoner DP, Gentile DA, Patel A, Doyle WJ (1999). Evidence for cytokine mediation of disease expression in adults experimentally infected with influenza A virus.. J Infect Dis.

[50] Svitek N, Rudd PA, Obojes K, Pillet S, von Messling V (2008). Severe seasonal influenza in ferrets correlates with reduced interferon and increased IL-6 induction.. Virology.

[51] Kobasa D, Takada A, Shinya K, Hatta M, Halfmann P (2004). Enhanced virulence of influenza A viruses with the haemagglutinin of the 1918 pandemic virus.. Nature.

[52] Pappas C, Aguilar PV, Basler CF, Solorzano A, Zeng H (2008). Single gene reassortants identify a critical role for PB1, HA, and NA in the high virulence of the 1918 pandemic influenza virus.. Proc Natl Acad Sci U S A.

[53] Salomon R, Franks J, Govorkova EA, Ilyushina NA, Yen HL (2006). The polymerase complex genes contribute to the high virulence of the human H5N1 influenza virus isolate A/Vietnam/1203/04.. J Exp Med.

[54] Vaillant L, La Ruche G, Tarantola A, Barboza P (2009). Epidemiology of fatal cases associated with pandemic H1N1 influenza 2009.. Euro Surveill.

[55] Rello J, Rodriguez A, Ibanez P, Socias L, Cebrian J (2009). Intensive care adult patients with severe respiratory failure caused by Influenza A (H1N1)v in Spain.. Crit Care.

