# Correction: Genomic Polymorphism of the Pandemic A (H1N1) Influenza Viruses Correlates with Viral Replication, Virulence, and Pathogenicity *In Vitro* and *In Vivo*

**DOI:** 10.1371/journal.pone.0235911

**Published:** 2020-07-06

**Authors:** Lili Xu, Linlin Bao, Jianfang Zhou, Dayan Wang, Wei Deng, Qi Lv, Yila Ma, Fengdi Li, Huihui Sun, Lingjun Zhan, Hua Zhu, Chunmei Ma, Yuelong Shu, Chuan Qin

Fig 3A is incorrect. The authors have provided a corrected version here.

**Fig 3:**
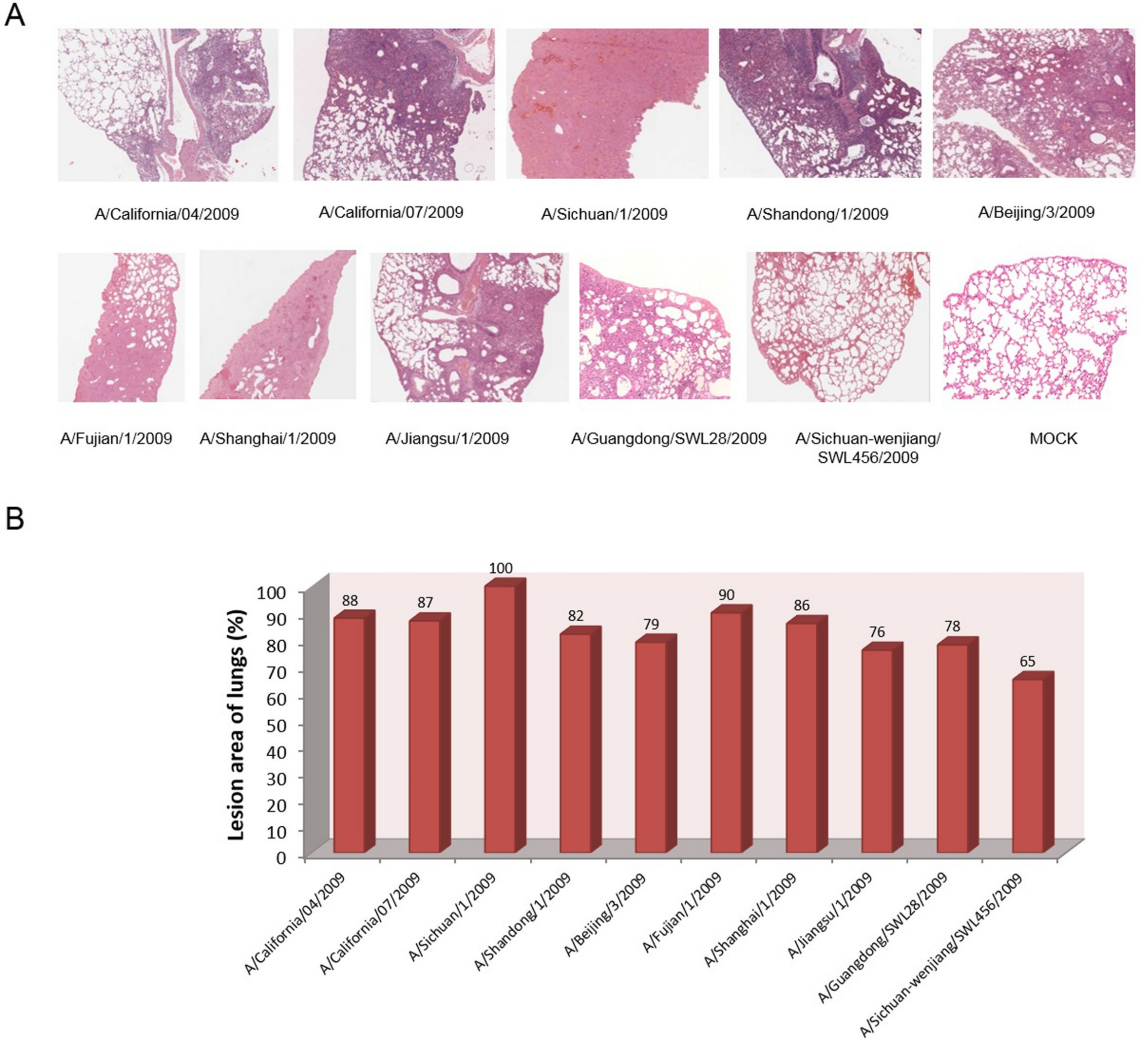
Pathological analysis of the lung tissues of challenged BALB/c mice. Ten mice from each group were euthanized at 5 d.p.i. to obtain lung tissue biopsies, and for each lung three 4-μm sections were stained with H&E for pathological investigations. (A) Representative sections of H&E stained lung tissues from 10^6^ TCID_50_ intranasally challenged mice. (B) Percentage of lesion area in lung tissues.
